# Smoking and microvascular free flap perfusion in head and neck reconstruction: radial free forearm flaps and anterolateral thigh flaps

**DOI:** 10.1038/s41598-022-18216-6

**Published:** 2022-08-16

**Authors:** Mark Ooms, Behrus Puladi, Khosrow Siamak Houschyar, Marius Heitzer, Ashkan Rashad, Johannes Bickenbach, Frank Hölzle, Ali Modabber

**Affiliations:** 1 Department of Oral and Maxillofacial Surgery, University Hospital RWTH Aachen, Pauwelsstraße 30, 52074 Aachen, Germany; 2 Department of Dermatology and Allergology, University Hospital RWTH Aachen, Pauwelsstraße 30, 52074 Aachen, Germany; 3 Department of Intensive Care Medicine, University Hospital RWTH Aachen, Pauwelsstraße 30, 52074 Aachen, Germany

**Keywords:** Reconstruction, Oral cancer

## Abstract

Head and neck reconstruction with microvascular free flaps is frequently performed in smokers. Smoking causes various alterations in the cardiovascular system. The aim of this study was to investigate the effects of smoking on flap perfusion as a critical factor for flap survival. A total of 370 patients reconstructed with a radial free forearm flap (RFFF) or anterolateral thigh flap (ALTF) in the head and neck region between 2011 and 2020 were retrospectively analyzed. Flap perfusion measurements with the O2C tissue oxygen analysis system were compared between nonsmokers, light smokers (< 20 pack-years), and heavy smokers (≥ 20 pack-years). The blood flow was intraoperatively equal in RFFFs (84.5 AU vs. 84.5 AU; *p* = 0.900) and increased in ALTFs (80.5 AU vs. 56.5 AU; *p* = 0.001) and postoperatively increased in RFFFs (114.0 AU vs. 86.0 AU; *p* = 0.035) and similar in ALTFs (70.5 AU vs. 71.0 AU; *p* = 0.856) in heavy smokers compared to nonsmokers. The flap survival rate was similar in nonsmokers, light smokers, and heavy smokers (97.3%, 98.4%, and 100.0%). Smoking partially increases rather than decreases microvascular free flap perfusion, which may contribute to similar flap survival rates in smokers and nonsmokers.

## Introduction

Microvascular free flaps are commonly used to reconstruct large defects in the head and neck region after ablative cancer surgery with an overall success rate of more than 95%^1–3^. In particular, the radial free forearm flap (RFFF) and the anterolateral thigh flap (ALTF) are frequently used, both of which have the advantages of variable size, constant anatomy, and a long pedicle^4–6^.

Flap survival is influenced by various patient and technical factors, one of which is smoking^7^. Smoking is a highly prevalent risk factor for head and neck cancer, and microvascular reconstruction of the head and neck region after ablative cancer surgery is thus often performed in smoking patients^2,8^. Smoking is associated with vascular pathologies, which can compromise flap perfusion^2,9,10^.

The impact of smoking on flap survival has been previously investigated, with most studies reporting no negative impact^8,11,12^. However, one study identified smoking as a negative predictor for microvascular free flap survival in breast reconstruction but not in head and neck reconstruction^13^. Moreover, in an animal study, smoking was found to be associated with higher flap failure^14^.

Interestingly, although flap perfusion is critical for flap survival, the effects of smoking on flap perfusion has not been adequately studied^15^. Only one study showed that smoking is associated with impaired perfusion of microvascular rectus abdominis flaps used for breast reconstruction in terms of reduced flap blood flow^16^.

It remains unclear, whether smoking affects microvascular free flap perfusion in the RFFF and ALTF used in head and neck reconstruction, and whether similar flap survival rates in smokers and nonsmokers reconstructed with microvascular free flaps in the head and neck region could be due to unimpaired flap perfusion in smokers.

Thus, this study aimed to investigate the influence of smoking on flap perfusion, a critical factor for flap survival.

## Results

### Comparison of clinical parameters between groups

The study population consisted of 370 patients: 222 nonsmokers, 63 light smokers, and 85 heavy smokers (Table 1).

**Table 1:** Characteristics of the study population.

Variable	NS (n = 222)	LS (n = 63)	*p* value	HS (n = 85)	*p* value
**Sex* (n)***					
Male	108 (48.6%)	39 (61.9%)	0.063	55 (64.7%)	**0.012**
Female	114 (51.4%)	24 (38.1%)		30 (35.3%)	
Age (years)	69.0 (20.0)	62.0 (14.0)	**< 0.001**	60.0 (11.0)	**< 0.001**
BMI (kg/m^2^)	25.0 (5.6)	24.6 (7.0)	0.157	22.5 (6.6)	**< 0.001**
**ASA* (n)***					
1 + 2	125 (56.3%)	30 (47.6%)	0.222	39 (45.9%)	0.101
3 + 4	97 (43.7%)	33 (52.4%)		46 (54.1%)	
***Flap type (n)***					
RFFF	127 (57.2%)	38 (60.3%)	0.659	43 (50.6%)	0.297
ALTF	95 (42.8%)	25 (39.7%)		42 (49.4%)	
***Flap location (n)***					
Tongue	37 (16.7%)	11 (17.5%)	**0.046**	11 (12.9%)	< 0.001*
Floor of mouth	30 (13.5%)	16 (25.4%)		37 (43.5%)	
Mandible	51 (23.0%)	10 (15.9%)		12 (14.1%)	
Maxilla + hard palate	30 (13.5%)	6 (9.5%)		9 (10.6%)	
Cheek	26 (11.7%)	6 (9.5%)		4 (4.7%)	
Soft palate	6 (2.7%)	6 (9.5%)		7 (8.2%)	
Extraoral	42 (18.9%)	8 (12.7%)		5 (5.9%)	
**Diabetes* (n)***					
No	182 (82.0%)	53 (84.1%)	0.693	78 (91.8%)	**0.033**
Yes	40 (18.0%)	10 (15.9%)		7 (8.2%)	
**Arterial hypertension *(n)***					
No	133 (59.9%)	40 (63.5%)	0.607	55 (64.7%)	0.440
Yes	89 (40.1%)	23 (36.5%)		30 (35.3%)	
**Peripheral arterial disease *(n)***					
No	218 (98.2%)	58 (92.1%)	0.028**	83 (97.6%)	0.670**
Yes	4 (1.8%)	5 (7.9%)		2 (2.4%)	
**Prior neck dissection* (n)***					
No	171 (77.0%)	53 (84.1%)	0.225	73 (85.9%)	0.086
Yes	51 (23.0%)	10 (15.9%)		12 (14.1%)	
**Prior neck irradiation* (n)***					
No	196 (88.3%)	60 (95.2%)	0.155**	76 (89.4%)	0.844
Yes	26 (11.7%)	3 (4.8%)		9 (10.6%)	
Surgery duration (min)	527.0 (189.0)	547.0 (169.0)	0.329	550.0 (153.0)	0.117
Duration of flap ischemia (min)	104.0 (38.0)	99.5 (39.0)	0.365	107.0 (30.0)	0.980

Parameters are indicated as numbers (with percentage) for categorical data (sex, ASA, flap type, flap location, diabetes, arterial hypertension, peripheral arterial disease, prior neck dissection, prior neck irradiation) or median (with interquartile range) for metric data (age, BMI, surgery duration, duration of flap ischemia) (separately described for the group of nonsmokers (NS), the group of light smokers (LS) and the group of heavy smokers (HS)); *p* values corresponding to testing for differences between groups (NS vs. LS and NS vs. HS) with chi-squared test (sex, ASA, flap type, flap location (NS vs. LS), diabetes, arterial hypertension, prior neck dissection, prior neck irradiation (NS vs. HS)), Freeman Halton test (*) (flap location (NS vs. HS)), Fisher’s exact test (**) (peripheral arterial disease, prior neck irradiation (NS vs. LS)) or Mann Whitney Test (age, BMI, surgery duration, duration of flap ischemia); significant *p* values are bold; *NS* nonsmokers, *LH* light smokers, *HS* heavy smokers, *BMI* body mass index, *ASA* American Society of Anesthesiologists score, *RFFF* radial free forearm flap, *ALTF* anterolateral thigh flap.

The light smokers and nonsmokers showed differences in age (*p* < 0.001), peripheral arterial disease (*p* = 0.028), and flap location (*p* = 0.046) (Table 1).

The heavy smokers and nonsmokers showed differences in sex (*p* = 0.012), age (*p* < 0.001), BMI (*p* < 0.001), diabetes (*p* = 0.033), and flap location (*p* < 0.001) (Table 1).

The flap revision rates were similar in light smokers and heavy smokers when separately compared with nonsmokers for all flaps (6.3% vs. 5.4%, *p* = 0.760; and 5.9% vs. 5.4%, *p* = 1.000), for RFFFs (5.3% vs. 6.3%, *p* = 1.000; and 9.3% vs. 6.3%, *p* = 0.501), and for ALTFs (8.0% vs. 4.2%, *p* = 0.603; and 2.4% vs. 4.2%, *p* = 1.000) (Table 2). Flap revision was performed for 8 RFFFs (8 venous congestions) and 4 ALTFs (2 venous congestions and 2 arterial insufficiencies) in nonsmokers, for 2 RFFFs (2 venous congestions) and 2 ALTFs (2 venous congestions) in light smokers, and for 4 RFFFs (4 venous congestions) and 1 ALTF (1 arterial insufficiency) in heavy smokers. The flap survival rates were similar in light smokers and heavy smokers when separately compared with nonsmokers for all flaps (98.4% vs. 97.3%, *p* = 1.000; and 100.0% vs. 97.3%, *p* = 0.192), for RFFFs (97.4% vs. 98.4%, *p* = 0.547; and 100.0% vs. 98.4%, *p* = 1.000), and for ALTFs (100.0% vs. 95.8%, *p* = 0.579; and 100.0% vs. 95.8%, *p* = 0.312). In nonsmokers, 2 RFFFs and 4 ALTFs were lost, and in light smokers, 1 RFFF was lost. No flap was lost in heavy smokers.

**Table 2:** Flap revision and survival.

Variable	NS (n = 222)	LS (n = 63)	*p* value	HS (n = 85)	*p* value
**Flap revision**					
**All flaps* (n)***					
No	210 (94.6%)	59 (93.7%)	0.760	80 (94.1%)	1.000
Yes	12 (5.4%)	4 (6.3%)		5 (5.9%)	
**RFFF* (n)***					
No	119 (93.7%)	36 (94.7%)	1.000	39 (90.7%)	0.501
Yes	8 (6.3%)	2 (5.3%)		4 (9.3%)	
**ALTF* (n)***					
No	91 (95.8%)	23 (92.0%)	0.603	41 (97.6%)	1.000
Yes	4 (4.2%)	2 (8.0%)		1 (2.4%)	
**Flap survival**					
**All flaps* (n)***					
No	6 (2.7%)	1 (1.6%)	1.000	0 (0.0%)	0.192
Yes	216 (97.3%)	62 (98.4%)		85 (100.0%)	
**RFFF* (n)***					
No	2 (1.6%)	1 (2.6%)	0.547	0 (0.0%)	1.000
Yes	125 (98.4%)	37 (97.4%)		43 (100.0%)	
**ALTF* (n)***					
No	4 (4.2%)	0 (0.0%)	0.579	0 (0.0%)	0.312
Yes	91 (95.8%)	25 (100.0%)		42 (100.0%)	

Parameters are indicated as numbers (with percentage) (separately described for the group of nonsmokers (NS), the group of light smokers (LS) and the group of heavy smokers (HS)); *p* values corresponding to testing for differences between groups (NS vs. LS and NS vs. HS) with Fisher’s exact test; *NS* nonsmokers, *LH* light smokers, *HS* heavy smokers, *RFFF* radial free forearm flap, *ALTF* anterolateral thigh flap.

### Comparison of flap perfusion parameters between groups

**Figure 1:**
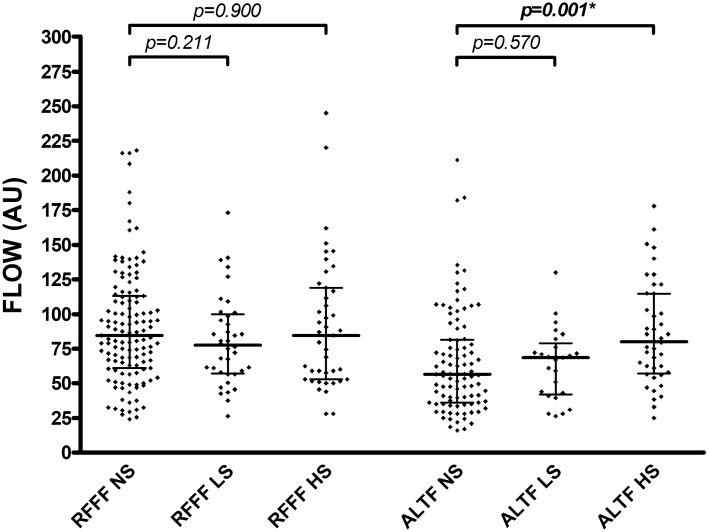
Intraoperative perfusion measurement. Each dot represents an individual blood flow (AU) with median (center line) and 1. and 3. quartile (upper and lower lines) for intraoperative measurement (separately described for the group of nonsmokers (NS), the group of light smokers (LS) and the group of heavy smokers (HS)); *p* values corresponding to testing for differences with Mann Whitney test; significant *p* values are bold (*p = 0.006 with adjustment for sex, age, BMI, diabetes, mean arterial pressure and catecholamine dose); *RFFF* radial free forearm flap, *ALTF* anterolateral thigh flap, *NS* nonsmokers, *LS* light smokers, *HS *heavy smokers, *AU*  arbitrary units.

**Figure 2:**
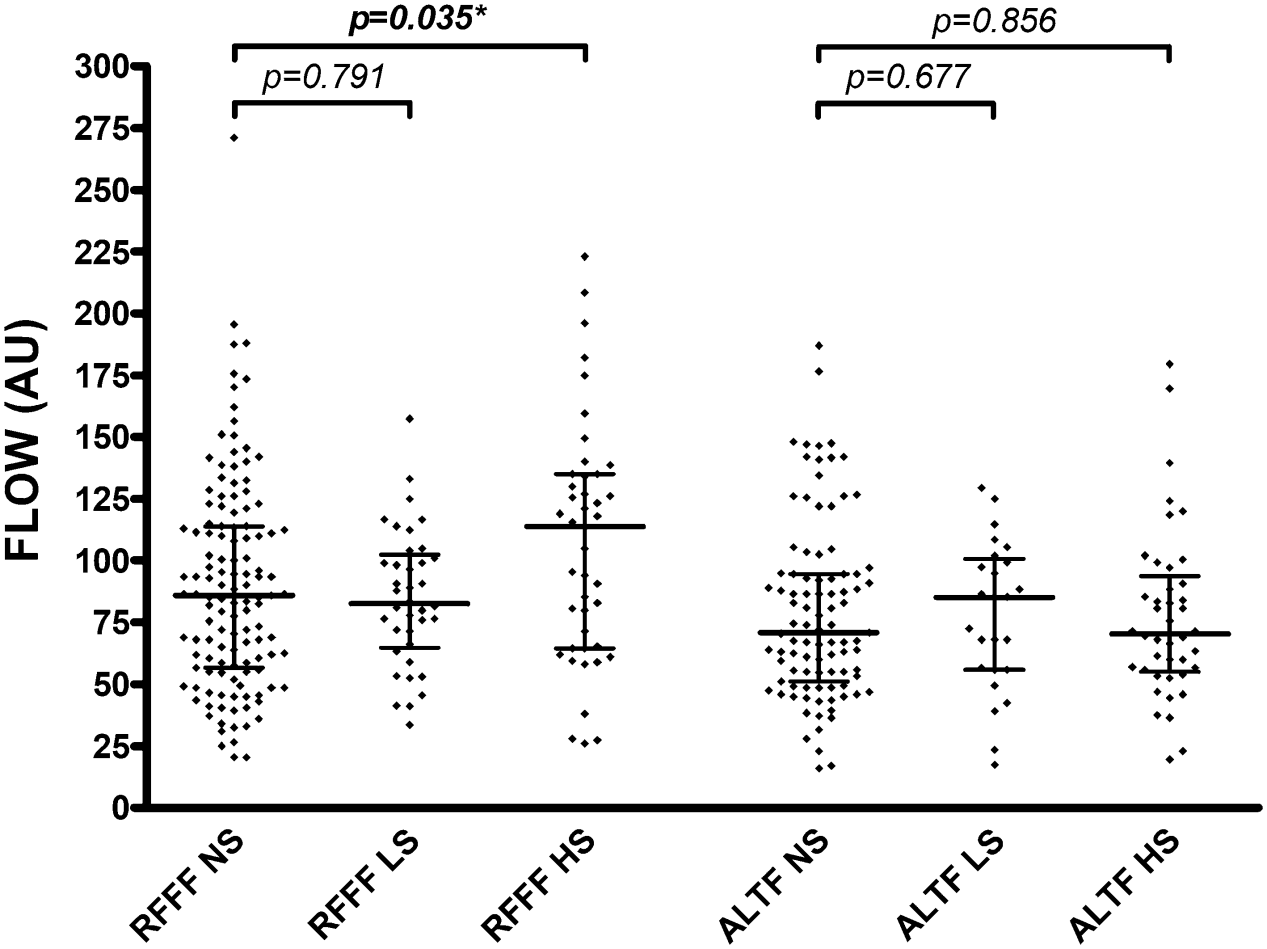
Postoperative perfusion measurement. Each dot represents an individual blood flow (AU) with median (center line) and 1. and 3. quartile (upper and lower lines) for postoperative measurement (separately described for the group of nonsmokers (NS), the group of light smokers (LS) and the group of heavy smokers (HS)); *p* values corresponding to testing for differences with Mann Whitney test; significant *p* values are bold (*p = 0.046 with adjustment for sex, age, BMI, diabetes, mean arterial pressure and catecholamine dose); *RFFF* radial free forearm flap, *ALTF* anterolateral thigh flap, *NS* nonsmokers, *LS* light smokers, *HS* heavy smokers, *AU* arbitrary units.

For RFFFs and ALTFs, light smokers and nonsmokers showed no differences in intraoperative flap blood flow (*p* = 0.211 and *p* = 0.570), hemoglobin concentration (*p* = 0.537 and *p* = 0.360), and hemoglobin oxygen saturation (*p* = 0.836 and *p* = 0.637) (Table 3, Fig. 1). In addition, no differences were observed between light smokers and nonsmokers for RFFFs and ALTFs in postoperative flap blood flow (*p* = 0.791 and *p* = 0.677), hemoglobin concentration (*p* = 0.837 and *p* = 0.286), and hemoglobin oxygen saturation (*p* = 0.094 and *p* = 0.434) (Table 3, Fig. 2).

**Table 3:** Flap perfusion parameters.

Variable	NS (n = 222)	LS (n = 63)	*p* value	HS (n = 85)	*p* value
**Intraoperative measurement**					
**RFFF (n = 208)**					
Blood flow (AU)	84.5 (52.0)	77.5 (41.5)	0.211	84.5 (66.0)	0.900
Hemoglobin [c] (AU)	58.0 (16.0)	57.8 (23.5)	0.537	57.5 (18.0)	0.588
Hemoglobin oxygen saturation (%)	77.0 (24.0)	79.3 (16.3)	0.836	80.5 (21.0)	0.997
**ALTF (n = 162)**					
Blood flow (AU)	56.5 (45.5)	68.5 (37.0)	0.570	80.5 (57.0)	0.001*
Hemoglobin [c] (AU)	42.5 (19.5)	46.0 (11.5)	0.360	48.5 (15.8)	0.167
Hemoglobin oxygen saturation (%)	57.5 (24.5)	60.0 (29.0)	0.637	60.8 (26.4)	0.452
**Postoperative measurement**					
**RFFF (n = 208)**					
Blood flow (AU)	86.0 (57.5)	82.5 (36.4)	0.791	114.0 (70.5)	0.035*
Hemoglobin [c] (AU)	53.5 (18.0)	53.8 (14.0)	0.837	54.0 (14.5)	0.946
Hemoglobin oxygen saturation (%)	70.0 (23.5)	61.0 (26.9)	0.094	68.0 (21.5)	0.957
**ALTF (n = 162)**					
Blood flow (AU)	71.0 (43.5)	85.0 (44.8)	0.677	70.5 (36.6)	0.856
Hemoglobin [c] (AU)	38.5 (17.0)	41.0 (16.8)	0.286	38.5 (11.9)	0.749
Hemoglobin oxygen saturation (%)	51.0 (24.5)	44.0 (30.0)	0.434	48.5 (21.6)	0.683

Parameters are indicated as median (with interquartile range) for intraoperative and postoperative measurement of RFFF and ALTF (separately described for the group of nonsmokers (NS), the group of light smokers (LS) and the group of heavy smokers (HS)); *p* values corresponding to testing for differences between groups (NS vs. LS and NS vs. HS) with Mann Whitney test; significant *p* values are bold (*p values < 0.05 with adjustment for sex, age, BMI, diabetes, mean arterial pressure and catecholamine dose); *NS* nonsmokers, *LH* light smokers, *HS* heavy smokers, *RFFF* radial free forearm flap, *ALTF* anterolateral thigh flap, *AU* arbitrary units, [*c*] concentration.

Heavy smokers showed equal intraoperative flap blood flow (84.5 AU vs. 84.5 AU; *p* = 0.900) for RFFFs and increased intraoperative flap blood flow (80.5 AU vs. 56.5 AU; *p* = 0.001) for ALTFs compared to nonsmokers (Table 3, Fig. 1). For RFFFs and ALTFs, no differences were observed in intraoperative hemoglobin concentration (*p* = 0.588 and *p* = 0.167) and hemoglobin oxygen saturation (*p* = 0.977 and *p* = 0.452) between heavy smokers and nonsmokers (Table 3). Heavy smokers showed increased postoperative flap blood flow (114.0 AU vs. 86.0 AU; *p* = 0.035) for RFFFs and similar postoperative flap blood flow (70.5 AU vs. 71.0 AU; *p* = 0.856) for ALTFs compared with nonsmokers (Table 3, Fig. 2). For RFFFs and ALTFs, no differences were observed for postoperative hemoglobin concentration (*p* = 0.946 and *p* = 0.749) and hemoglobin oxygen saturation (*p* = 0.957 and *p* = 0.683) between heavy smokers and nonsmokers (Table 3).

After adjusting for sex, age, BMI, diabetes, mean arterial blood pressure, and catecholamine dose, the difference in intraoperative flap blood flow for ALTFs and postoperative flap blood flow for RFFFs between heavy smokers and nonsmokers persisted (*p* = 0.006 and *p* = 0.046).

Flap perfusion parameters described separately for 2 mm and 8 mm measurement depth, data for the regressions analysis including flap location, and data for testing differences between intraoperative and postoperative measurements of blood flow are added as supplementary data (Supplementary Table 1, 2 and 3).

## Discussion

This study aimed to investigate the potential influence of smoking on microvascular free flap perfusion in head and neck reconstruction. Smoking is a patient factor of increasing interest, as there is a high prevalence of smoking patients undergoing reconstruction with microvascular free flaps in the head and neck region after ablative cancer surgery, and smoking has been linked to cardiovascular changes that may affect flap perfusion, a critical factor for flap survival^2,8–10,15^. Previous studies have mainly focused on the relationship between smoking and flap survival without considering flap perfusion^8,11–13^. Only one study investigated the influence of smoking on the flap perfusion of microvascular rectus abdominis free flaps used for breast reconstruction, reporting a decrease in flap blood flow in smoking patients^16^. It remains unclear whether smoking affects microvascular free flap perfusion in the RFFF and ALTF used in head and neck reconstruction, and whether similar flap survival rates in smokers and nonsmokers could be due to unimpaired flap perfusion in smokers.

This study followed commonly used definitions for nonsmokers and smokers^17–19^. The smokers were stratified into heavy smokers—exposure of 20 or more pack-years—and light smokers—exposure of less than 20 pack-years—to account for the cumulative chronic pathophysiological effects of smoking^20^. The O2C tissue oxygen analysis system was used as an established and noninvasive method to measure various microcirculatory parameters, such as flap blood flow, flap hemoglobin concentration, and flap hemoglobin oxygen saturation^15,21,22^. All the flaps had perfusion parameters in both measurements indicating flap viability and were therefore representative of sufficiently perfused microvascular free flaps (blood flow: RFFF ≥ 20 AU and ALTF ≥ 15 AU; hemoglobin oxygen saturation: RFFF ≥ 15% and ALTF ≥ 10%)^23^.

This study is the first to show that perfusion of microvascular free flaps is partially increased in smokers, as the flap blood flow was increased in ALTFs intraoperatively and in RFFFs postoperatively in heavy smokers compared with nonsmokers. These differences persisted when adjusting for both between-group differences in sex, age, BMI, and diabetes, and for the presumed factors that determine flap blood flow, such as mean arterial blood pressure and catecholamine dose^24^. As the flap location was assumed to exert no effect on flap blood flow, no adjustment was made for it. No differences in flap blood flow were observed between light smokers and nonsmokers.

In light of smoking-induced cardiovascular changes, such as an induction of intima fibrosis with consequently higher vascular stiffness and vasoconstriction, both of which presumably can reduce flap perfusion, the observation of increased rather than decreased flap blood flow in heavy smokers is unexpected^8,10^. However, regarding the cardiovascular effects of smoking, different mediators and pathophysiological mechanisms must be considered^25^. The short-term cardiovascular effects of smoking, such as the reduction of dermal perfusion due to nicotine-mediated vasoconstriction, were unlikely to be present at the time of measurements, as patients were abstinent from smoking since the preoperative night, and nicotine’s half-life in humans is approximately 2 h^25^. However, smoking is associated with longer-lasting short term pathophysiological effects, such as decreased aerobe metabolism and attenuated inflammatory response, which are mitigated by smoking cessation of more than 4 weeks^26^. The long-term cardiovascular effects of smoking, such as oxygen radical-mediated vascular stiffening, were likely to be present at the time of measurements, but it remains unclear whether these cardiovascular changes, which presumably reduce flap blood flow, are even pronounced, or at least significantly pronounced, in the region of interest, the vessels used for anastomosis and the flap tissue. Interestingly, one study showed that the dermal perfusion of the distal forearm as the donor site for the RFFF and the thigh as the donor site for the ALTF were similar in smokers and nonsmokers^27^. These results are consistent with our findings to the extent that flap perfusion did not differ between light smokers and nonsmokers. Nevertheless, compared to nonsmokers, flap blood flow was even increased in heavy smokers in the ALTF intraoperatively and in the RFFF postoperatively. The increased ALTF blood flow in heavy smokers contrasts with the findings of a study that showed that blood flow in microvascular rectus abdominis flaps was reduced intraoperatively up to four hours after reperfusion^16^. Although they are both perforator flaps, the differences between the rectus abdominis flap and ALTF in terms of blood flow cannot be excluded. The higher blood flow in ALTF intraoperatively and in the RFFF postoperatively may be related to the absence of the nicotine-mediated vasoconstrictive effect in heavy smokers at the time of measurement because of smoking abstinence^25,28^. The different timing of this process, early in ALTF and later in RFFF, is consistent with the observations in two methodically comparable studies that after anastomosis to the cervical recipient vessels, blood flow increases earlier in ALTF than in RFFF, and may be due to the different anatomic characteristics of the flaps, as the RFFF is a fasciocutaneous flap with multiple small vessels supplying the skin and the ALTF is a perforator flap with a single or at least a low number of perforators supplying the skin^23,29–31^. Interestingly, only in heavy smokers did blood flow values in ALTF decrease absolutely from intraoperative to postoperative measurement, which may reflect a delayed onset of counter regulation to the proposed effect of the absence of the nicotine-mediated vasoconstrictive effect. However, internal control measurements that could shed more light on this are missing.

In summary, smoking-related cardiovascular changes do not reduce microvascular free flap perfusion. Therefore, similar or even increased microvascular free flap perfusion in smokers might be a prerequisite for similar flap survival in smokers, as flap perfusion is a critical factor for flap survival^15^.

For both flap types, the revision rate and survival rate in this study were comparable to the literature, with a value below 10% and above 95%, respectively, and did not differ between nonsmokers, light smokers, and heavy smokers^3,32^. Smoking is known to increase the risk of thromboembolic events, so smokers were expected to have higher flap revision rates and lower flap survival rates^33,34^. However, several other studies have also shown that smoking has no effect on flap survival^8,11–13^.

Despite the inclusion of a large study population from a high-volume center and the use of an established measurement method, this study has limitations. It should be mentioned that a moist environment, owing to salivary flow and wound secretion, can aggravate the measurements, and that skin temperature, as skin perfusion depends on skin temperature, also affects the measurements^35,36^. An influence on the measurement by the moist environment is particularly pronounced for intraoral flaps; however, flap blood flow did not differ between intraoral flaps and extraoral flaps. Nevertheless, the skin surface of the flap was cleaned and dried before the measurements, and all measurements were performed in the operating theater or on the intensive care unit with room climate control, which attenuates the skin temperature differences between patients. However, it cannot be excluded that differences in skin temperature influenced the perfusion measurements. In addition, because of the retrospective design of the study and the classification of patients into nonsmokers and smokers based on clinical records, it was not possible to assess their time of smoking abstinence, which might affect our results because the groups of smokers were heterogenous in this aspect and an influence of acute effects of smoking on perfusion cannot be excluded^26^. In general, the differences between patients in vascular anatomy, such as vessel length and diameter, cannot be excluded, all of which may possibly affect flap perfusion.

This study offers insights to further understand the perfusion of microvascular free flaps and shows, for the first time, that smoking-related physiological changes do not reduce the perfusion of microvascular free flaps. It supports the conclusion that smoking should not be a general contraindication for microvascular free flap reconstruction and reinforces the traditional approach of favoring free flaps over pedicled flaps in smokers because of their better perfusion with less likelihood of partial necrosis, despite higher donor site morbidity when a free flap is used^37^. However, surgical complications that may be affected by smoking independently of flap perfusion and survival, such as wound healing disorders and infections, were not considered in this study. Therefore, preoperative smoking cessation or at least temporary abstinence from smoking, since it benefits patient outcomes by reducing surgical complications, is still recommended^38^. Further longitudinal studies are needed to confirm our results over time.

## Conclusion

Smoking has only a minor and smoking-amount-dependent impact on microvascular free flap perfusion in RFFF and ALTF. Increased or at least unrestricted flap blood flow could contribute to similar flap survival rates in smoking and nonsmoking patients. However, this should not affect the general recommendation for preoperative smoking cessation.

## Methods

### Study population

The study was approved by the local ethics committee of the Medical Faculty of RWTH Aachen University (EK 309-20). The local ethics committee of the Medical Faculty of RWTH Aachen University allowed us to waive informed consent for this human study. All methods were in accordance with the relevant guidelines and regulations.

The study population comprised 370 patients: 222 nonsmokers and 148 smokers who underwent reconstruction with a microvascular soft tissue free flap (RFFF or ALTF) in the head and neck region for malignant and nonmalignant disease at our Department of Oral and Maxillofacial Surgery between 2011 and 2020. Consistent with the commonly used definitions for smokers and nonsmokers, the patients were classified as smokers if they had ever smoked daily for at least a 6-month period and as nonsmokers if they had no smoking history at all or had not smoked daily at least a 6-month period^18^. The smokers were then divided in terms of the amount of smoking into light smokers, whose pack-year score was less than 20, and heavy smokers, whose pack-year score was 20 or more^17,19^. Patient’s comorbidities were recorded if the diagnosis was confirmed according to the discipline-specific guidelines. Prior neck dissection status was defined as neck dissection with an anatomic dissection of the recipient vessel later used for microvascular anastomosis in a separate operation before free tissue transfer, and prior neck irradiation status was defined as irradiation of the recipient vessel later used for microvascular anastomosis before free tissue transfer. Surgery duration was defined as the time between the first incision and the last suture, and flap ischemia duration was defined as the time between the termination of flap perfusion at the donor site and the onset of flap perfusion after release of the anastomosis at the recipient site. The surgical revision of the anastomosis with return to the operating room was defined as flap revision, and complete loss of the flap with removal of the flap by the time of hospital discharge was defined as flap failure. The data obtained from clinical records and operation reports were retrospectively analyzed. The patients with incomplete records or invalid measurements were excluded.

An Allen test was performed to verify adequate blood flow to the hand through the ulnar artery before harvesting each RFFF, and an acoustic Doppler examination was performed to locate perforator vessels in the anterolateral thigh region before harvesting each ALTF. Surgical procedures were performed under general anesthesia, and the flaps were raised according to a standard method routinely performed at our department^31^. The flap pedicle artery and the pedicle concomitant veins were anastomosed to the cervical arterial vessel system in an end-to-end configuration for the arterial vessels and in an end-to-end or end-to-side configuration for the venous vessels. Postoperative management included postoperative monitoring in the intensive care unit for at least one day with sedation until the morning of the first postoperative day. Invasive arterial blood pressure measurement with an arterial catheter was performed, and blood pressure regulation with a target systolic blood pressure above 125 mmHg was maintained overnight by central venous catecholamine (norepinephrine) administration, if necessary, at least until the next morning, after measuring the postoperative flap perfusion. For seven days, 5000 IU of heparin was injected intracutaneously three times daily.

### Measurement of perfusion parameters

The flaps were measured intraoperatively immediately after the completion and release of the anastomosis and postoperatively on the morning of the first postoperative day using the O2C tissue oxygen analysis system (O2C Oxygen-to-see, LEA Medizintechnik, Giesen, Germany)^21,39^.

The O2C device combines the techniques of laser Doppler spectroscopy and white light spectroscopy to determine blood flow (arbitrary units (AU)), hemoglobin concentration (AU), and hemoglobin oxygen saturation (%)^21^. Laser light (830 nm; 30 mW) and white light (500–800 nm; 50 W) are transmitted into the tissue, and the backscattered light is subsequently collected by the probe^39^. The blood flow is calculated by analyzing the Doppler shift of the laser light caused by the movement of the erythrocytes in the blood vessels as the product of erythrocyte quantity and velocity^39^. The hemoglobin concentration is calculated by analyzing the sum of absorbance of all the wavelengths of white light^39^. The hemoglobin oxygen saturation is calculated by analyzing the color change of white light compared with pre-recorded hemoglobin spectra with defined oxygen saturation, since hemoglobin is the main absorber in tissue and, as oxygenated hemoglobin, has two absorption peaks at 542 nm and 577 nm and, as deoxygenated hemoglobin, only one absorption peak at 566 nm^21,39^. The measurements mainly reflect capillary-venous oxygenation, as vessels larger than 100 µm in diameter completely absorb transmitted light, and 85% of hemoglobin is located in the capillary-venous compartment of microcirculation^21^.

The intraoperative and postoperative measurements were performed centrally on the cleaned and dried skin part of the flap, with the probe sealed with a sterile cover and a measurement interval of 10 s with 4-s lead time under ambient light compensation control. The values for 2 mm and 8 mm tissue depth were used to calculate the mean values for flap blood flow, hemoglobin concentration, and hemoglobin oxygen saturation. During measurement, invasive arterial blood pressure measurement was performed using an arterial catheter, and blood pressure regulation with a target systolic pressure above 125 mmHg was ensured by intravenous catecholamine administration.

### Statistical analysis

The patients were divided into a group of nonsmokers, a group of light smokers (< 20 pack-years), and a group of heavy smokers (≥ 20 pack-years). The patients were also divided into two classes according to the American Society of Anesthesiologists score (ASA): a group with an ASA score greater than 2, and a group with an ASA score less than or equal to 2. The differences between the group’s clinical parameters were analyzed using the chi-squared test, Freeman Halton test, and Fisher’s exact test for categorical data, and the Mann Whitney test for metric data (metric data was not normally distributed). The differences in flap blood flow, hemoglobin concentration, and hemoglobin oxygen saturation between groups were analyzed in univariable testing using the Mann Whitney test and the differences in flap blood flow between intraoperative and postoperative measurement were analyzed in univariable testing using the Wilcoxon signed rank test. Multivariable testing using multiple linear regression models with adjustment for sex, age, BMI, diabetes, mean arterial blood pressure, and catecholamine dose was performed with and without including flap location to determine significant differences in flap blood flow, hemoglobin concentration, and hemoglobin oxygen saturation between the groups after univariable testing. *p* values were two-tailed and considered statistically significant < 0.05. Statistical analysis was performed using SPSS version 26 (SPSS, IBM, New York, USA).

## Supplementary Information

Supplementary Tables.

## Data Availability

The datasets generated and analyzed during the current study are available from the corresponding author on reasonable request.
