# Supplementary material for: Smoking and microvascular free flap perfusion in head and neck reconstruction: radial free forearm flaps and anterolateral thigh flaps
Source: Sci Rep. 2022 Aug 16;12:13902. doi: 10.1038/s41598-022-18216-6 (PMC9381556; doi:10.1038/s41598-022-18216-6)
Supplement: Supplementary file 1 — Supplementary Tables. [file 41598_2022_18216_MOESM1_ESM.docx]

**Supplementary table 1: Flap perfusion parameters**

| **Variable** | **NS (n=222)** | **LS (n=63)** | **HS (n=85)** |
| --- | --- | --- | --- |
| **Intraoperative measurement** | | | |
| **RFFF (n=208)** | | | |
| ***Blood flow (AU) 2mm*** | 34.0 (32.0) | 26.5 (29.5) | 30.0 (38.0) |
| ***Blood flow (AU) 8mm*** | 130.0 (80.0) | 121.0 (54.8) | 134.0 (89.0) |
| ***Hemoglobin [c] (AU) 2mm*** | 71.0 (24.0) | 73.5 (26.8) | 69.0 (22.0) |
| ***Hemoglobin [c] (AU) 8mm*** | 45.0 (20.0) | 41.0 (29.3) | 45.0 (20.0) |
| ***Hemoglobin oxygen saturation (%) 2mm*** | 80.0 (28.0) | 86.0 (19.8) | 81.0 (25.0) |
| ***Hemoglobin oxygen saturation (%) 8mm*** | 79.0 (27.0) | 76.5 (28.3) | 80.0 (23.0) |
| **ALTF (n=162)** | | | |
| ***Blood flow (AU) 2mm*** | 16.0 (15.0) | 17.0 (9.5) | 27.5 (30.5) |
| ***Blood flow (AU) 8mm*** | 92.0 (73.0) | 105.0 (75.5) | 126.5 (88.3) |
| ***Hemoglobin [c] (AU) 2mm*** | 53.0 (25.0) | 60.0 (19.0) | 64.0 (25.5) |
| ***Hemoglobin [c] (AU) 8mm*** | 33.0 (19.0) | 32.0 (11.0) | 34.5 (11.0) |
| ***Hemoglobin oxygen saturation (%) 2mm*** | 57.0 (35.0) | 66.0 (21.5) | 61.5 (44.3) |
| ***Hemoglobin oxygen saturation (%) 8mm*** | 59.0 (25.0) | 56.0 (33.0) | 57.5 (32.0) |
| **Postoperative measurement** | | | |
| **RFFF (n=208)** | | | |
| ***Blood flow (AU) 2mm*** | 35.0 (41.0) | 39.5 (32.0) | 43.0 (47.0) |
| ***Blood flow (AU) 8mm*** | 125.0 (81.0) | 125.0 (57.3) | 154.0 (98.0) |
| ***Hemoglobin [c] (AU) 2mm*** | 65.0 (24.0) | 62.0 (15.3) | 66.0 (21.0) |
| ***Hemoglobin [c] (AU) 8mm*** | 42.0 (18.0) | 44.5 (21.0) | 40.0 (18.0) |
| ***Hemoglobin oxygen saturation (%) 2mm*** | 74.0 (25.0) | 70.0 (25.3) | 70.0 (20.0) |
| ***Hemoglobin oxygen saturation (%) 8mm*** | 71.0 (32.0) | 59.5 (31.3) | 63.0 (26.0) |
| **ALTF (n=162)** | | | |
| ***Blood flow (AU) 2mm*** | 25.0 (30.0) | 22.0 (26.5) | 27.5 (28.8) |
| ***Blood flow (AU) 8mm*** | 113.0 (76.0) | 131.0 (75.0) | 113.5 (50.0) |
| ***Hemoglobin [c] (AU) 2mm*** | 46.0 (24.0) | 51.0 (29.0) | 46.0 (21.3) |
| ***Hemoglobin [c] (AU) 8mm*** | 31.0 (12.0) | 31.0 (15.0) | 31.5 (11.3) |
| ***Hemoglobin oxygen saturation (%) 2mm*** | 49.0 (39.0) | 55.0 (22.5) | 55.0 (29.3) |
| ***Hemoglobin oxygen saturation (%) 8mm*** | 58.0 (31.0) | 48.0 (40.0) | 47.0 (33.5) |

Parameters are indicated as median (with interquartile range) for intraoperative and postoperative measurement of RFFF and ALTF (separately described for 2mm and 8mm depth and for the group of nonsmokers (NS), the group of light smokers (LS) and the group of heavy smokers (HS)); abbreviations: NS=nonsmokers, LH=light smokers, HS=heavy smokers, RFFF=radial free forearm flap, ALTF=anterolateral thigh flap, AU=arbitrary units, [c]=concentration.

**Supplementary table 2: Regression analysis (including flap location)**

| **Variable** | **Comparison** |  | **p-value** |
| --- | --- | --- | --- |
| **Intraoperative blood flow in ALTFs** | | | |
| ***Smoking*** | NS vs. HS |  | 0.006 |
| **Postoperative blood flow in RFFFs** | | | |
| ***Smoking*** | NS vs. HS |  | 0.080 |

P-value corresponding to multiple regression analysis for testing differences in blood flow between nonsmokers (NS) and heavy smokers (HS) upon adjustment for sex, age, BMI, diabetes, mean arterial pressure, catecholamine dose and flap location (intraoral vs. extraoral) (separately for intraoperative blood flow in ALTFs and postoperative blood flow in RFFFs); abbreviations: ALTF=anterolateral thigh flap, RFFF=radial free forearm flap, NS=nonsmokers, HS=heavy smokers.

**Supplementary table 3: Differences between intraoperative and postoperative blood flow**

| **Variable** | **IOP** | **POP** | **p-value** |
| --- | --- | --- | --- |
| **RFFF** | | | |
| ***Blood flow (AU) NS*** | 84.5 (52.0) | 86.0 (57.5) | 0.950 |
| ***Blood flow (AU) LS*** | 77.5 (41.5) | 82.5 (36.4) | 0.215 |
| ***Blood flow (AU) HS*** | 84.5 (66.0) | 114.0 (70.5) | 0.050 |
| **ALTF** | | | |
| ***Blood flow (AU) NS*** | 56.5 (45.5) | 71.0 (43.5) | <0.001 |
| ***Blood flow (AU) LS*** | 68.5 (37.0) | 85.0 (44.8) | 0.071 |
| ***Blood flow (AU) HS*** | 80.0 (57.0) | 70.5 (36.6) | 0.088 |

Parameters are indicated as median (with interquartile range) for intraoperative and postoperative measurement of mean blood flow (separately described for RFFFs and ALTFs and for the group of nonsmokers (NS), the group of light smokers (LS) and the group of heavy smokers (HS)); p-values corresponding to testing for differences between intraoperative and postoperative measurement with Wilcoxon signed rank test; abbreviations: IOP=intraoperative measurement, POP=postoperative measurement, NS=nonsmokers, LH=light smokers, HS=heavy smokers, RFFF=radial free forearm flap, ALTF=anterolateral thigh flap, AU=arbitrary units.
